# Supplementary material for: A confinable home-and-rescue gene drive for population modification
Source: eLife. 2021 Mar 5;10:e65939. doi: 10.7554/eLife.65939 (PMC7968924; doi:10.7554/eLife.65939)
Supplement: Supplementary file 1. [file elife-65939-supp1.docx]

**Supplementary File 1.** Target sequence of gRNA and primers used in this study.

| Name | Primer sequence (5’ – 3’) |
| --- | --- |
| *gRNA#1^PolG2^* | GCGCAGCCGGGACACAAGGCTGG |
| *gRNA#2^PolG2^* | GACTGATGCAGTTGCGCAGCCGG |
| 1073A.S1F | CCGAGCTATCTGCTGACTTGTCTGATC |
| 1073A.S2R | TGGAAGGTTCCGCTAGGCTGCAGTGC |
| de*PolG2* gBlock | CGGACTGATGCAGTTGCGCAGCCGGGACACAcGcCTcGCcGAaACgATcCAtATtAGtGAcGTgCCcGAtTAcTTgTTgAAcATcTTcAAgAAtTAgTTTAAACTGAATCGTTTTTAAAATAACAAATCAATTGTTTTATAATATTCGTACGATTCTTTGATTATGTAATAAAATGTGATCATTAGGAAG |
| ExuL.1F | CTCGACGGTCACGGCGGGCATGTCGACGCGGCCGCCTGTCATCTCTGGCGCAACCATTTG |
| ExuL.2R | TCTCCGTCGTGGTCCTTATAGTCCATCTCGAGTTTCTTATATGATCTGCGAAAAGCAATC |
| 1095.C1F | CGACGGTCACGGCGGGCATGTCGACGCGGCCGCGCAGACATAGACGAGCAACAATCAATC |
| 1095.C2R | GTCTCCGTCGTGGTCCTTATAGTCCATCTCGAGCGCAAAGCTGGTTTTTTTTCGCGTTAG |
| βTub.1F | CGACGGTCACGGCGGGCATGTCGACGCGGCCGCTCGACCCATCCCATTATACACCCATAT |
| βTub.2R | GTCTCCGTCGTGGTCCTTATAGTCCATCTCGAGTTTGATAGTAAAGTTAGGGCCCCTTTT |
| 1076B.S9F | CCTTTCCACAAAGCCAGCAATTCCGTAACAATTTCC |
| 1076B.S2R | CATGGGTTTATTTGCAACAAACACAATTGTGGCG |
| 1076B.S3F | CTCTGCTTCTGGATGGCTGTGATCATGG |
| 1076B.S4R | CAGACAAGTCAGCAGATAGCTCGGAATCAC |
| 1073A.S1F | TGAATGCAATTGTTGTTGTTAACTTGTTTATTGCAGCTT |
| 1076B.S10R | GCGGAGACACAGCGCATCCTGGGATCTGAC |
| 1076B.S7F | CGATCGATGCTAAAGGATTTGGGTCCC |
| 1076B.S8R | GCCAGCCACAATGCCGCCAAGCAGGAC |
| 1073A.S3F | CACTTGCTGGAAACGGATATGCTCGGC |
| 1073A.S4R | GGCAAGGTGGTGGGCTTTAATGTCCG |
